# Clinical Features and Body Composition in Men with Hormone-Sensitive Metastatic Prostate Cancer: A Pilot Study Examining Differences by Race

**DOI:** 10.1155/2022/9242243

**Published:** 2022-06-02

**Authors:** Patricia M. Sheean, Paula O'Connor, Cara Joyce, Vasilios Vasilopoulos, Ami Badami, Melinda Stolley

**Affiliations:** ^1^Loyola University Chicago, 2160 South First Avenue, Cuneo 439, Maywood, Chicago, IL 60153, USA; ^2^Loyola University Chicago, 2160 South First Avenue, Building 115, Room 254, Maywood, Chicago, IL 60153, USA; ^3^Loyola University Medical Center, 2160 South First Avenue, Maywood, Chicago, IL 60153, USA; ^4^Medical College of Wisconsin, Division of Hematology and Oncology, Milwaukee, WI 53226, USA

## Abstract

Black men treated with frontline therapies for metastatic prostate cancer (MPC) show better clinical outcomes than non-Black men receiving similar treatments. Variations in body composition may contribute to these findings. However, preliminary data are required to support this concept. We conducted a retrospective cohort study for all men with MPC evaluated at our center over a 4-year period, collecting demographic and clinical data (*N* = 74). Of these, 55 men had diagnostic computed tomography images to quantify adipose tissue and skeletal muscle, specifically sarcopenia and myosteatosis. Nineteen men had repeat imaging to explore changes over time. Frequencies, medians, interquartile ranges, and time to event analyses (hazard ratios (HR); confidence interval (CI)) are presented, stratified by race. Overall, 49% (*n* = 27) of men had sarcopenia, 49% (*n* = 27) had myosteatosis, and 29% (*n* = 16) had sarcopenia and myosteatosis simultaneously. No significant relationship between body mass index (Log-rank *p*=0.86; HR: 1.05, 95% CI: 0.45–2.49) or sarcopenia (Log-rank*p*=0.92; HR: 1.01, 95% CI: 0.46–2.19) and overall survival was observed. However, the presence of myosteatosis at diagnosis was associated with decreased overall survival (Log-rank *p*=0.09; HR: 2.34, 95% CI: 1.05–5.23), with more pronounced (statistically nonsignificant) negative associations for Black (HR: 4.39, 95% CI: 0.92–21.1, *p*=0.06) versus non-Black men (HR: 1.89, 95% CI: 0.79–4.54, *p*=0.16). Over the median 12.5 months between imaging, the median decline in skeletal muscle was 4% for all men. Black men displayed a greater propensity to gain more adipose tissue than non-Black men, specifically subcutaneous (*p*=0.01). Because of the potential for Type II errors in this pilot, future studies should seek to further evaluate the implications of body composition on outcomes. This will require larger, adequately powered investigations with diverse patient representation.

## 1. Introduction

Prostate cancer (PC) is the most frequently diagnosed malignancy among men, displaying the highest incidence and lowest survival for Black men when compared to men of other races/ethnicities [1]. Although most men with PC are diagnosed with localized disease, 10–20% of men will initially present with *de novo* metastatic prostate cancer (MPC) [2, 3]. This condition is showing incremental increases each year in developed countries [4–6]. MPC reflects PC that has spread beyond the prostate gland, most commonly to the bones, distant lymph nodes, liver, and other organs [7]. While this disease is highly treatable due to evolving treatment options, it remains incurable. Frontline treatment for hormone-sensitive MPC involves androgen deprivation therapy (e.g., antiandrogens and gonadotropin-releasing hormone agonists) with or without chemotherapy (e.g., docetaxel) [8]. According to Smith et al., chemotherapy is used more often for patients with greater disease burden and good performance status [9]. Sole treatment with antihormonal therapies is offered to patients with lower performance status and/or a preference for oral over intravenous therapies [9]. Interestingly, despite presenting with higher risk disease profiles, Black men treated with frontline therapies for MPC show better clinical outcomes than White men receiving similar treatments [10–12].

Sarcopenia (i.e., *reductions in skeletal muscle (SM) mass*) and myosteatosis (i.e., *fatty infiltrated SM*) are considered adverse body composition phenotypes associated with compromised nutritional status and metabolic alterations [13, 14]. Within the broader context of metastatic cancer, these conditions have emerged as predictors of chemotherapy toxicity, shorter time to tumor progression (TTP), and decreased overall survival (OS) [15]. Evidence from a recent review on body composition in the MPC setting [16] supports associations between low levels of SM, increased disease progression, and shorter OS for men with MPC on antiandrogen therapies [17–19] and on single-agent chemotherapy [20–22]. Simultaneously, Xu et al. report that high body mass index (BMI) predicts longer survival in men with MPC, independent of sarcopenia or myosteatosis [23]. Collective limitations of this work entail the predominant inclusion of non-Hispanic White men or not considering race as an effect modifier of outcomes. Thus, variations in body composition may contribute to the differential outcomes in Black versus non-Black men observed in previous studies [10–12]. This has yet to be considered in previous analyses. Therefore, we sought to explore differences in body composition in a pilot cohort of men undergoing initial treatment for MPC. Based on observations in diverse healthy populations [24, 25], we hypothesized that Black men would exhibit a higher prevalence of sarcopenia and lower levels of visceral adipose tissue (VAT) than non-Black men at diagnosis.

## 2. Materials and Methods

We conducted a retrospective cohort study for all men with MPC who presented to Loyola University Medical Center from February 2016 to February 2020. This study was approved by the Institutional Review Board of Loyola University Chicago. Names and medical record numbers were generated from the hospital cancer registry. This institutional database contains specific and relevant information (e.g., cancer type/subtype, stage, date of diagnosis, and date of death, if applicable) for patients evaluated at our center. Staging was recorded in the database, in accordance with the American Joint Committee on Cancer TMN system. Study personnel screened patients on this list using the following eligibility criteria: adult male (≥18 years), any race/ethnicity, and histologically confirmed prostate cancer (Stage IVb).

### 2.1. Clinical Data

Demographic (e.g., self-reported race, marital status, and insurance) and clinical information (e.g., presenting symptomology, medical therapies, laboratory values, smoking status, height, weight, and radiology reports) were obtained from the electronic health record (EHR). All patients received androgen deprivation therapies (androgen receptor inhibitors, gonadotropin-releasing hormone analogues, or gonadotropin-releasing hormone antagonists) with or without chemotherapy (docetaxel). Patients were selected for inclusion in our study if they were diagnosed with hormone-sensitive MPC and had not received any other frontline systemic therapies in the metastatic setting. The duration of follow-up was the number of months from confirmed diagnosis to the date of death noted in the hospital cancer registry, EHR, or posted publicly. If date of death was uncertain, men were right censored at their last known contact. Updates on progression and survival concluded in January 2021.

### 2.2. Body Composition Image Analysis

To evaluate body composition, archived computed tomography (CT) images were requested from the Department of Radiology for eligible patients. Diagnostic images were used to assess pretreatment body composition. If available, the successive CT image was used to assess body composition change. A Radiology Technician accessed the Picture Archiving and Communication System to retrieve the specified test and locate the axial slice inclusive of the third lumbar (L3) region. All images were saved in Digital Imaging and Communications in Medicine (DICOM) format on an encrypted research network and subsequently analyzed by trained personnel using Slice-O-Matic (v 4.3, Tomovision, Montreal, QB). Automated tissue demarcation with manual correction was conducted using Hounsfield unit (HU) thresholds specific to the tissue compartment (−29 to +150 for SM, −190 to −30 for subcutaneous adipose tissue (SAT), and −150 to−50 for VAT) [26–28]. Quality assurance was performed on 10 random images by an outside expert (Dr. Sandra Gomez-Perez) certified in body composition analyses and not involved in data collection. Images were discussed and reanalyzed, if needed. Cross-sectional areas (cm^2^) of the SM and adipose tissue compartments were computed and normalized for stature to derive skeletal muscle index (SMI, cm^2^/m^2^), VAT Index (VATI, cm^2^/m^2^), and SAT Index (SATI, cm^2^/m^2^).

### 2.3. Definitions

Sarcopenia was classified by applying the BMI-specific cut-points for males proposed by Martin et al. (BMI <25.0 and L3 SMI <43 cm^2^/m^2^ or BMI ≥25.0 and L3 SMI <53 cm^2^/m^2^) [29]. Additionally, sarcopenic obesity was defined as a combination of BMI ≥30 kg/m^2^ and L3 SMI <53 cm^2^/m^2^. SM radiodensity (expressed as mean HU) was used to categorize myosteatosis using the sex-specific and BMI-specific cut-points of Martin et al. (<41 SMHU for BMI <24.9 kg/m^2^ and <33 SMHU for BMI ≥25.0 kg/m^2^) [29]. Clinical outcomes included time to tumor progression (time elapsed from diagnosis to biochemical and/or radiologic evidence of disease, as noted by the treating physician and change of therapy) and overall survival (time elapsed from diagnosis to date of death). Toxicities were assessed and analyzed after the first two cycles of docetaxel. This window reflects a time when dose reductions or dose suspensions (occurrences used to convey clinical significance) are needed for subsequent cycles and when disease progression is less likely to occur.

### 2.4. Statistical Analysis

Data analyses were completed using SAS (version 9.4; Cary, NC). Descriptive statistics were performed to examine differences in baseline demographics, clinical features, and body composition characteristics stratified by race (Black versus non-Black). Patient characteristics are presented as medians, interquartile ranges, or counts and percentages. Statistical differences between groups were assessed with Wilcoxon rank-sum tests or Fisher's exact test for continuous and nominal variables, respectively. Univariable comparisons of time to event outcomes (TTP; OS) were performed using Kaplan–Meier curves and log-rank tests. Main effects and interaction terms for race and body composition variables were assessed in age-adjusted Cox proportional hazards models to estimate hazard ratios (HRs) and corresponding 95% confidence intervals (CIs). A *p* value of <0.05 was used to denote statistical significance.

## 3. Results

In total, 110 patients were screened from the cancer center registry over the 4-year time span. Of these, 36 patients were ineligible (i.e., not Stage IVb, diagnostic imaging conducted at an outside hospital, and simultaneous cancer diagnoses (e.g., lung and lymphoma)). For the remaining 74 men with clinical data, 55 had available/evaluable baseline CT imaging, and of these, 19 had repeat images to permit comparisons across time. In general, participants presented at a median age of 71 years with overweight (median BMI of 27.6 kg/m^2^) and a median of 2 comorbid conditions, of which 28% had diabetes. As depicted in Table 1, differences in clinical features at diagnosis were noted. Specifically, Black men presented with lower hemoglobin (*p*=0.04), higher creatinine (*p*=0.05), lower albumin (*p*=0.001) levels, higher prevalence of diabetes (*p*=0.05), and more weight loss (*p*=0.05) than non-Black men. In the subsample with CT imaging (*n* = 55) (Table 2), 49% (*n* = 27) had sarcopenia, and 49% (*n* = 27) had myosteatosis. Overall, 17 patients (31%) had neither sarcopenia nor myosteatosis, 11 (20%) had sarcopenia without myosteatosis, 11 (20%) had myosteatosis without sarcopenia, and 16 (29%) had sarcopenia and myosteatosis simultaneously. When applying the normal, overweight, or obese categorizations, BMI did not statistically differ across racial strata. Black men displayed lower VAT (73.5 versus 199.8 cm^2^, *p*=0.04), which remained significantly lower than non-Black men after adjusting VAT for height (VAT Index 25.1 versus 73.7 cm^2^/m^2^, *p*=0.04). Although SAT appears higher in Black versus non-Black men (188.6 versus 146.4 cm^2^, *p*=0.40), this was not significantly higher until adjusted for height (SAT Index 56.3 versus 51.6 cm^2^/m^2^, *p*=0.04). When SM quality (SMHU) and adipose tissue quality (VATHU, SATHU, and IMATHU) were examined, no differences emerged across groups. Toxicity leading to dose reductions occurred in 28% (*n* = 7/25) of participants on docetaxel; only 1 individual was Black.

In a small subset of patients with repeat imaging (*n* = 19; 26% Black), we explored body composition changes over time (Table 3). The median time between diagnostic to subsequent imaging was 12.5 months. The overall median decline in SM was 4% for all men. Although not statistically significant, Black men experienced a 2% SMI gain, while non-Black men displayed a 7% SMI loss. In this subset, we also examined the incidence of sarcopenia. Of the eight men with repeat imaging who were not sarcopenic at baseline, none developed sarcopenia at the time of follow-up. Black men appear to gain adipose tissue (SAT, VAT, and IMAT) more readily than non-Black men, showing significant differences in SAT changes (+95.2 versus +23.2 cm^2^ or 8% versus 5% median gain, *p*=0.01, resp.). No differences were noted for SM or adipose tissue quality radiodensities across time.

**Figure 1:**
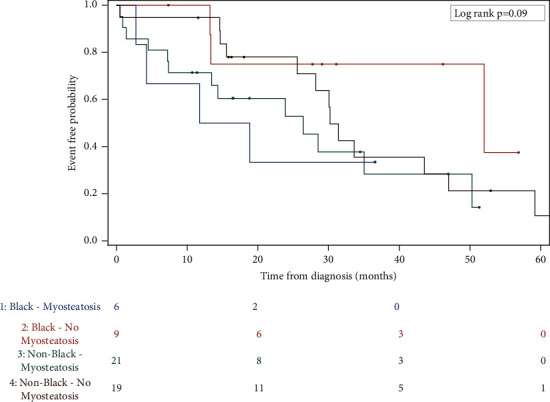
Overall survival by myosteatosis stratified by race.

Time to event analyses demonstrated no significant relationship between BMI (Log-rank *p*=0.86; HR: 1.05, 95% CI: 0.45–2.49) or sarcopenia (Log-rank *p*=0.92; HR: 1.01, 95% CI: 0.46–2.19) and OS. However, the presence of myosteatosis at diagnosis was associated with decreased OS (Log-rank *p*=0.09; HR: 2.34, 95% CI: 1.05–5.23), showing more pronounced (statistically nonsignificant) negative associations for Black (HR: 4.39, 95% CI: 0.92–21.1, *p*=0.06) versus non-Black men (HR: 1.89, 95% CI: 0.79–4.54, *p*=0.16) (Figure 1).

## 4. Discussion

Body composition represents a collection of tissue biomarkers consisting of SM and adipose tissue with potential prognostic value. Due to technological advances in the last decade or so, quantifying body composition using image-based technologies has become relatively easier, including the availability of public or commercial analytical software [30]. As a result, we now appreciate the adverse associations between SM abnormalities on cancer-specific outcomes in host of tumor types across the globe [15, 31–33]. For men with early-stage prostate cancer, the inverse relationship between antiandrogen therapies, declines in SM health, and increased adiposity is well accepted [34]. By design, antiandrogen treatments cause an abrupt inhibition of testosterone production. This, in turn, has a direct, negative impact on protein synthesis with implications for the hypogonadal-obesity cycle. This complex pathophysiological process involves endocrine, neuronal, and adipokine aberrations [35]. Overall, men with MPC are disproportionally underrepresented in studies examining the prevalence and implications of body composition abnormalities [15]. Men with MPC reflect an especially vulnerable patient population, as they are more likely to present with obesity (an independent risk factor for PC) or depleted levels of SM. These conditions may then be further exacerbated by frontline treatments known to promote SM decline, as well as weight gain [36]. Thus, our pilot findings contribute to the overall limited literature on men with MPC and provide novel data on body composition by race.

At diagnosis, about half of our participants were sarcopenic, and half displayed myosteatosis. Considering that the prevalence of sarcopenia was not different between groups, we were unable to accept our hypothesis that sarcopenia would be higher among Black versus non-Black men. Our prevalence findings are lower than those of Xu et al. (53% sarcopenia and 59% myosteatosis) [23] and Stangl-Kremser et al. (83% sarcopenia; myosteatosis not investigated) [21] and in line with Cushen et al. (47% sarcopenia; myosteatosis not investigated) [20]. Sarcopenia is considered a progressive and generalized SM disorder, long associated with aging and increased risk of falls, frailty, fractures, and disability. Interestingly, body composition data from the National Health and Nutrition Examination Survey shows SM deterioration and the risk of sarcopenia has nearly tripled among Blacks in a relatively short period of time (6.2% in 1999–2000 to 20.6% in 2005–2006; *p* for trend <0.001), regardless of age [24]. The definition of sarcopenia has recently been revised to highlight the additional importance of identifying this condition in clinical populations and measuring muscle function (strength and physical performance) to subvert adverse outcomes [37]. Inherent in any retrospective study relying on CT imaging, only one dimension of sarcopenia is captured, that is, reduced SM mass. However, low levels of SM mass alone are clinically significant within oncology patients, as they equate to a depleted volume of cytotoxic drug distribution [38]. Patients with sarcopenia have an increased propensity to display serious toxicities often necessitating dose reductions or drug suspensions [38]. This concept has been demonstrated in colorectal [39, 40], renal [41–43], and thyroid cancers [44], as well MPC (on docetaxel) [20]. Treatment disruptions contribute to the associations between sarcopenia and decreased OS. Of the patients in our study who received frontline docetaxel (*n* = 25/74), 32% (*n* = 8) experienced dose-limiting toxicities, a finding similar to Cushen et al. (35%; *n* = 22/63) [20]. Due to small numbers, we were unable to explore associations with SM. We find it intriguing that the majority of men who did experience toxicities were non-Black. Given our findings regarding the higher amounts of SM at baseline (adjusted for height) among Black men, this merits investigation in future studies.

Myosteatosis is an umbrella term used to describe varying adipose compartments within the SM (intermuscular and intramuscular adipose tissue and intramyocellular lipids). Myosteatosis occurs more often in individuals with diabetes [45], increases with antiandrogen therapy [46], and is a negative prognostic indicator of OS in MPC [23, 47]. Based on the magnitude of the HRs and the visual representation of the curves in Figure 1, our pilot data signals myosteatosis may be a particularly worrisome condition for Black men. In general, the pathophysiology of myosteatosis is not well appreciated, and differences in race/ethnicity have received limited consideration [48]. However, it is well accepted that this condition is more prevalent in aging muscle, and it reflects insulin resistance and decreased muscle function [14]. In animal models, accumulating evidence suggests that myosteatosis may symbolize mitochondrial dysregulation and decreased fatty acid oxidation [49, 50], which are exacerbated by excess nutrient supply and obesity. At present, very few studies have targeted strategies to improve fatty infiltrated muscle [51, 52]. Given current models supporting as synergistic relationship between hyperinsulinemia and carcinogenesis [53], caloric restriction and weight loss seem essential components of any future intervention targeting improved SM and cancer-related outcomes.

In general, Black men shoulder a disproportionate PC burden, displaying the highest incidence and mortality rates compared to other races/ethnicities [1]. Higher rates of comorbidities, such as obesity, diabetes, and cardiovascular disorders, are thought to negatively affect survival rates for men with PC. In addition, biological (e.g., genetic susceptibility) and socioenvironmental factors (e.g., stress, racism, and healthcare access) are also implicated [54]. Yet, it is unclear if these assumptions apply to the MPC setting. Recent data support superior clinical outcomes, specifically prostate-specific antigen response and overall survival, for Black versus non-Black men receiving frontline therapies [10–12]. In our sample, Black men did appear to possess more adverse clinical features at diagnosis, as evidenced by their laboratory abnormalities, diabetes history, and propensity for weight loss when compared to non-Black men. Although BMI classifications did not show statistical differences between racial strata (*p*=0.19), Black men had nearly two times the prevalence of obesity (BMI ≥30.0 kg/m^2^) than non-Black participants (46% versus 26%, resp.). Interestingly, two studies have shown that, among men with MPC, obesity better predicts docetaxel tolerance [22] and is associated with longer OS, independent of sarcopenia or myosteatosis [23]. However, in the study by Wu et al. [22], sarcopenia was nearly nonexistent (*n* = 3/333 men), and only 14% of participants were non-Caucasian in the investigation by Xu et al. [23]. While our study sample is relatively small, the lower prevalence of sarcopenia at diagnosis, the time to event analyses regarding myosteatosis and OS, and the propensity to retain SM over time collectively support that body composition may be of particular importance for Black versus non-Black men, regardless of obesity. Given the more pathologic role of visceral adipose tissue on metabolic abnormalities and overall mortality [55], the lower levels of VAT at baseline for Black versus non-Black men (*p*=0.04) are also intriguing and in keeping with our proposed hypothesis. Taken together, these findings support that more work is needed to elucidate the biological mechanisms underlying or reflective of these relationships. They also underscore the essential nature of identifying more diverse study cohorts.

This pilot study explored potential differences in clinical features and body composition by race among men with MPC. Several limitations are worth noting. First, by design, this study sample is small and likely underpowered to detect true relationships that might exist having a larger, representative population of men with metastatic PC been sampled (i.e., Type II error). Second, we lack information on important factors not found in the EHR. Our conventional approach has been to collect “real-time” data to complement our retrospective findings or to generate novel data on lifestyle behaviors (e.g., quality of life and health habits), nutritional status, symptom burden, and life stressors to inform intervention development. Unfortunately, due to restrictions imposed by the global pandemic, this was not possible. Lifestyle behaviors, specifically nutrition and physical activity, can favorably alter body composition and are key targets for improving PC recurrence and mortality [56]. Information within diverse MPC populations remains very limited. Third, we applied the definitions of Martin et al. to define sarcopenia and myosteatosis [29]. While employed widely in this field, these cut-points were developed in predominantly non-Hispanic White patient populations. The implications of race remain unknown. Finally, to maximize sample size, we grouped all non-Black men as a comparator group, applying the methodologies of Smith et al. [9], and used self-reported race data found in the EHR. This approach may require further consideration, especially genetic ancestry, if a larger, more diverse sample is available.

## 5. Conclusions

Our pilot findings contribute to the limited literature regarding body composition in men diagnosed with MPC. For men with MPC, sarcopenia and myosteatosis are highly prevalent conditions. In our study, about half the men had sarcopenia or myosteatosis, although 30% had neither. Importantly, they provide novel data on differences in body composition by race linked to relevant outcomes.

- At diagnosis, Black men demonstrated significantly lower levels of visceral adipose tissue (*p*=0.04) and higher levels of subcutaneous adipose tissue (adjusted for height) (*p*=0.04) than non-Black men.
- The presence of myosteatosis at diagnosis is associated with decreased OS (Log-rank *p*=0.09; HR: 2.34, 95% CI: 1.05–5.23), showing more pronounced negative associations for Black versus non-Black men.
- Over the course of treatment, Black men displayed a greater propensity for increased adiposity than non-Black men, specifically subcutaneous adipose tissue (*p*=0.01).

Because of the propensity for Type II errors in this pilot, future studies should seek to further evaluate the implications of body composition on short-and long-term relevant clinical outcomes. This will require larger, adequately powered investigations with diverse patient representation.

## Figures and Tables

**Table 1:** Clinical features in Black versus non-Black men with hormone-sensitive metastatic prostate cancer^ab^.

	Overall *N* = 74	Non-Black *n* = 51 (69%)	Black *n* = 23 (31%)	*p* value
Age at diagnosis (yr), median (IQR)	71 (63–79)	71 (63–81)	70 (65–75)	0.9441
<65 yr, *n* (%)	23 (31)	16 (31)	7 (30)	1.000
≥65 yr, *n* (%)	51 (69)	35 (69)	16 (70)	
Married, *n* (%)	43 (58)	33 (65)	10 (44)	0.2969
Insurance, *n* (%) (*n* = 73)				0.3203
Private	41 (56)	29 (58)	12 (52)	
Public	31 (43)	21 (42)	10 (44)	
Smoking status, *n* (%) (*n* = 72)				0.4024
Current	3 (4)	1 (2)	2 (9)	
Former	40 (56)	28 (56)	12 (55)	
Never	29 (40)	21 (42)	8 (36)	
Body mass index (kg/m^2^), median (IQR) (*n* = 71)	27.6 (24.8–31.1)	28.3 (25.2–31.0)	26.3 (22.5–32.8)	0.5907
ECOG at diagnosis, *n* (%)				0.0589
0	59 (80)	44 (86)	15 (65)	
≥1	15 (20)	7 (14)	8 (35)	
Baseline PSA (ng/mL), median (IQR) (*n* = 51)	110 (35–677)	81 (22–403)	304 (53–1402)	
Gleason score, *n* (%)				0.1736
7	27 (36)	16 (31)	11 (48)	
8–10	47 (64)	35 (69)	12 (52)	
Total distant metastases, median (IQR)	1 (1-2)	1 (1-2)	2 (1-2)	0.8065
Bone metastases, *n* (%)	56 (76)	39 (76)	17 (74)	1.000
Laboratory values, median (IQR)				
Hemoglobin (g/dL) (*n* = 71)	12.5 (11.0–13.69)	13.0 (11.5–13.9)	11.1 (8.6–13.7)	0.0408
Lymphocytes (10^9^ cells/liter) (*n* = 65)	1.4 (1.0–1.8)	1.3 (1.0–1.8)	1.6 (1.2–1.8)	0.5270
Creatinine (mg/dL) (*n* = 73)	(1.10.05)(0.9–1.6)	1.1 (0.9–1.4)	1.5 (1.1–1.7)	0.0454
ALT (U/L) (*n* = 70)	21 (15–27)	21 (15–29)	18 (15–22)	0.2722
ALP (U/L) (*n* = 70)	95 (68–229)	89 (65–216)	117 (73–412)	0.4456
Albumin (g/dL) (*n* = 70)	3.6 (3.4–4.1)	3.7 (3.5–4.2)	3.4 (3.1–3.6)	0.0055
Comorbid conditions, median (IQR)	2 (1–3)	2 (1–3)	2 (2-3)	0.4455
History of diabetes, *n* (%)	21 (28)	11 (22)	10 (43)	0.0530
Presenting symptoms, *n* (%)				
None	7 (9)	7 (14)	0 (0)	0.0912
Fatigue	11 (15)	6 (12)	5 (22)	0.3008
Hematuria	18 (24)	12 (23)	6 (26)	0.8124
Lower urinary tract	45 (61)	32 (63)	13 (57)	0.6118
Pain	28 (38)	18 (35)	10 (43)	0.5017
Weight loss	21 (28)	11 (22)	10 (43)	0.0530
Frontline docetaxel, *n* (%)	25 (34)	18 (35)	6 (26)	0.4336
Progression, *n* (%) (*n* = 62)^c^	34 (55)	26 (60)	8 (44)	0.2928
Died, *n* (%) (*n* = 66)	36 (55)	26 (56)	10 (50)	0.6248

^a^ALT = alanine aminotransferase, ALP = alkaline phosphatase, ECOG = European Cooperative Oncology Group; IQR = interquartile range, and PSA = prostate-specific antigen. ^b^Median follow-up for all patients 23.3 (13.4–35.0), Black 18.9 (11.8–36.5), and non-Black 24.7 (14.6–35.0) in months. ^c^Sample less than 74 as progression status was unknown for 12 men.

**Table 2:** Body composition characteristics in Black versus non-Black men with treatment naïve hormone-sensitive metastatic prostate cancer^a^.

	Overall *N* = 55	Non-Black *n* = 40	Black *n* = 15	*p* value
Body weight, kg (median, IQR)	88.0 (74.8–99.2)	85.6 (75.1–93.9)	92.1 (64.5–103.6)	0.3119
Body mass index category, kg/m^2^				0.1923
18.5–24.9 kg/m^2^, *n* (%)	12 (22)	8 (20)	4 (27)	
25.0–29.9 kg/m^2^, *n* (%)	25 (46)	21 (54)	4 (27)	
≥30.0 kg/m^2^, *n* (%)	17 (32)	10 (26)	17 (46)	
Sarcopenia,^b^*n* (%)	27 (49)	22 (55)	5 (33)	0.1523
Sarcopenic obesity,^c^*n* (%)	8 (15)	6 (15)	2 (13)	1.0000
Skeletal muscle index, cm^2^/m^2^ (median, IQR)	49.8 (40.2–56.2)	49.7 (39.8–54.3)	56.2 (47.3–62.7)	0.0682
Skeletal muscle HU (median, IQR)	33.8 (26.3–41.8)	32.5 (26.9–42.6)	35.5 (23.1–43.9)	0.6707
Myosteatosis^d^ (*n*, %)	27 (49)	21 (53)	6 (40)	0.4089
Visceral adipose tissue (cm^2^) (median, IQR)	187.5 (106.5–302.2)	199.8 (147.4–296.7)	73.5 (53.5–319.3)	0.0441
Visceral adipose tissue index (cm^2^/m^2^) (median, IQR)	61.6 (33.6–98.9)	73.7 (46.9–102.6)	25.1 (17.5–93.1)	0.0441
Visceral adipose tissue HU (median, IQR)	−86.9 [(−93.3)–(−81.4)]	−88.3 [(−93.3)–(−83.6)]	−81.5 [(−96.0)–(−73.6)]	0.2450
Subcutaneous adipose tissue (cm^2^) (median, IQR)	162.0 (99.2–216.3)	146.4 (97.9–206.7)	188.6 (103.7–243.6)	0.3899
Subcutaneous adipose tissue index (cm^2^/m^2^) (median, IQR)	51.9 (33.3–69.4)	51.6 (33.2–65.9)	56.3 (35.1–80.5)	0.0440
Subcutaneous adipose tissue HU (median, IQR)	−92.1 [(−99.6)–(−84.0)]	−94.9 [(−99.5)–(−85.1)]	−89.5 [(−100.9)–(−65.5)]	0.2730
Intramuscular adipose tissue (cm^2^) (median, IQR)	18.1 (13.1–23.8)	18.3 (13.3–23.0)	17.3 (10.8–25.5)	0.9022
Intramuscular adipose tissue HU (median, IQR)	−36.7 [(−46.3)–(29.1)]	−39.3 [(−50.2)–(28.6)]	−34.7 [(−40.0)–(30.6)]	0.2301

^a^HU = Hounsfield units and IQR = interquartile range. ^b^Sarcopenia defined as skeletal muscle index <43 for BMI <25.0 kg/m^2^ and skeletal muscle index <53 for BMI ≥25.0 kg/m^2^ per the cut-points of Martin et al. [29]. ^c^Sarcopenic obesity defined as a combination of BMI ≥30 kg/m^2^ and L3 SMI <53 cm^2^/m^2^. ^d^Myosteatosis defined as <41 SMHU for BMI <24.9 kg/m^2^ and <33 SMHU for BMI ≥25.0 kg/m^2^ per the cut-points of Martin et al. [29].

**Table 3:** Changes in body composition for Black versus non-Black men with hormone-sensitive metastatic prostate cancer and repeat computed tomography imaging^a^.

	Non-Black *n* = 14	Black *n* = 5	*p* value
Skeletal muscle index change, cm^2^/m^2^ (median, IQR)	−3.7 [(−8.5)–(−0.4)] (7% loss)	−1.2 [(−1.8)–12.5)] (2% gain)	0.2870
Subcutaneous adipose tissue change (cm^2^) (median, IQR)	23.2 [(−6.5)−51.5] (5% gain)	95.2 (89.4–172.3) (8% gain)	**0.0109**
Visceral adipose tissue change (cm^2^) (median, IQR)	−16.0 [(−32.2)−3.5] (1% gain)	40.1 (29.4–41.0) (2% gain)	**0.0577**
Intramuscular adipose tissue change (cm^2^) (median, IQR)	3.1 (0.4–5.6) (10% gain)	8.4 (6.7–11.7) (21% gain)	**0.0710**
Skeletal muscle HU change (median, IQR)	−5.7 [(−9.6)−(3.2)] (14%)	−5.3 [(−7.2)−(−1.4)] (12%)	0.6770
Subcutaneous adipose tissue HU change (median, IQR)	−5.0 [(−9.6]−(−3.2)]	−8.0 [(−7.2)−(1.4)]	0.2114
Visceral adipose tissue HU change (median, IQR)	−0.6 [(−6.0)−(1.6)]	−1.6 [(−4.5)−4.4]	0.8896
Intramuscular adipose tissue HU change (median, IQR)	−3.8 [(−10.6)−(0.2)]	−7.4 [(−14.5)−(1.7)]	0.8170

^a^HU = Hounsfield and IQR = interquartile range.

## Data Availability

Data used to support the findings of this study are restricted by the LUC Institutional Review Board to protect patient privacy. Data are available from Dr. Sheean (psheean1@luc.edu) for researchers who meet the criteria for access to confidential data.

## Abbreviations

BMI: Body mass index
CT: Computed tomography
CI: Confidence interval
DICOM: Digital imaging and communications in medicine
EHR: Electronic health record
HR: Hazard ratios
HU: Hounsfield unit
IMAT: Intramuscular adipose tissue
IMATHU: Intramuscular adipose tissue Hounsfield unit
OS: Overall survival
PC: Prostate cancer
MPC: Metastatic prostate cancer
SM: Skeletal muscle
SMHU: Skeletal muscle Hounsfield unit
SMI: Skeletal muscle index
SAT: Subcutaneous adipose tissue
SATHU: Subcutaneous adipose tissue Hounsfield unit
SATI: Subcutaneous adipose tissue index
L3: Third lumbar region
TTP: Time to tumor progression
VAT: Visceral adipose tissue
VATHU: Visceral adipose tissue Hounsfield unit
VATI: Visceral adipose tissue index.
